# Large Tuberculosis Outbreaks — United States, 2017–2023

**DOI:** 10.15585/mmwr.mm7516a1

**Published:** 2026-04-30

**Authors:** Kala M. Raz, Maryam B. Haddad, Sandy P. Althomsons, Lauren Cowan, J. Steve Kammerer, Chee Kin Lam, Clinton J. McDaniel, James Posey, Sarah Talarico, William L. Walker, Noah G. Schwartz, Jonathan M. Wortham

**Affiliations:** 1 Division of Tuberculosis Elimination, National Center for HIV, Viral Hepatitis, STD, and TB Prevention, CDC.

**Summary**

**What is already known about this topic?**

During 2014–2016, a total of 24 large tuberculosis (TB) outbreaks (10 or more related TB cases within a 3-year period) were identified within the United States, primarily affecting U.S.-born persons.

**What is added by this report?**

During 2017–2023, a total of 50 large TB outbreaks were identified in 23 states, primarily involving U.S.-born persons. Persons with TB in large outbreaks reported substance use, homelessness, and incarceration more often than did other persons with TB. Two thirds of large outbreaks occurred within family and social networks.

**What are the implications for public health practice?**

Nationwide capacity for outbreak detection, prevention, and response is critical for reducing outbreak-associated morbidity. Building trust within affected communities and overcoming barriers to diagnosis and treatment associated with homelessness and substance use are critical for outbreak prevention.

## Abstract

During 2017–2023, based on an analysis of national genomic and tuberculosis (TB) case surveillance data, 50 large TB outbreaks (10 or more related TB cases in a 3-year period) involving 1,092 cases were identified in 23 states. Compared with 61,993 other persons who received a diagnosis of TB during this period, persons included in large outbreaks were more frequently U.S.-born (79% versus 26%), and a higher percentage reported substance use (27% versus 12%), homelessness (9% versus 5%), and incarceration (11% versus 3%). Approximately one fourth of these large outbreak-related cases were identified through contact tracing; these cases less commonly had clinical markers of highly infectious disease (23%) than did large outbreak-related cases identified through other methods (including evaluation associated with symptoms, targeted testing, or incidental findings) (61%), suggesting that contact tracing might have facilitated earlier diagnosis. Among the 50 large outbreaks, 34 (68%) were primarily associated with family or social networks, and 13 (26%) were primarily associated with congregate settings. Maintaining state and local public health capacity for outbreak detection, prevention, and response is essential, even in low-incidence jurisdictions. Effective outbreak responses must overcome barriers to diagnosis and treatment associated with homelessness and substance use and include efforts to build trust with affected communities. Procedures to promptly identify and isolate persons with infectious TB remain critical in congregate settings.

## Introduction

Although the United States has one of the lowest incident tuberculosis (TB) rates in the world (*1*), TB outbreaks still occur. Interrupting TB transmission requires prompt diagnosis and treatment of both TB disease and asymptomatic latent TB infection (LTBI), which can progress to infectious TB disease if not treated. Health departments play a central role in these activities by providing treatment for persons with TB disease, which typically involves ≥4 months of directly observed therapy, and contact tracing to identify and evaluate persons exposed to *Mycobacterium tuberculosis* so that those with TB disease or LTBI can be treated. When barriers limit these core public health activities, transmission can continue, and outbreaks can occur (*2*–*5*).

In 2014, to better characterize TB outbreaks and guide national public health strategies for outbreak prevention and response, CDC began nationwide surveillance for large TB outbreaks, defined as 10 or more related TB cases in a 3-year period (*2*). During 2014–2016, a total of 24 large outbreaks comprising 518 total cases were identified, primarily involving U.S.-born persons, with transmission within both households and nonfamily social networks (*2*). To continue to characterize large outbreaks and understand the populations affected, this report analyzes and describes large TB outbreaks identified in the United States during 2017–2023, the most recent years for which data are available.

## Methods

### Data Sources

All TB cases diagnosed in the United States are reported to the National Tuberculosis Surveillance System, which collects data on patient demographic and clinical characteristics, medical and social risk factors, and epidemiologic links to other cases. Since 2018, CDC has sponsored whole genome sequencing of *M. tuberculosis* complex isolates from all persons with culture-positive TB disease.^†^ Information about settings and networks of transmission was obtained during routine communications with state and local health departments. These data were summarized using a standardized form, then shared with health departments, which were given an opportunity to confirm or correct the information.

### Large Outbreak Identification and Case Inclusion

Using national genomic and case surveillance data, CDC’s Division of Tuberculosis Elimination defines large TB outbreaks as those for which 10 or more verified cases of TB^§^ related by transmission occur within a 3-year period (*2*). Cases are considered related by transmission if their *M. tuberculosis* isolates differ by five or fewer single nucleotide polymorphisms or, when sequencing data are unavailable (e.g., for cases diagnosed without culture confirmation), an epidemiologic link^¶^ to another outbreak-related case is present. Genetically or epidemiologically linked cases that occur after identification of a large outbreak continue to be classified as outbreak related until a 2-year period elapses with two or fewer cases identified.

### Descriptive Analysis

This analysis included large TB outbreaks identified in the 50 U.S. states and the District of Columbia during 2017–2023. Outbreaks described in this report can include cases reported during 2014–2023, although some outbreaks were still ongoing as of 2023.** The analysis excluded two large outbreaks caused by surgical implantation of contaminated bone allografts because these outbreaks did not represent person-to-person transmission and required distinct prevention and response strategies (*6*). Demographic, clinical, and social or behavioral characteristics of persons with TB in large outbreaks were compared with those of all other U.S. persons with TB reported during 2017–2023. These characteristics included substance use, defined as self-reported alcohol use to excess,^††^ injection drug use, or noninjection drug use within the year preceding diagnosis; experiencing homelessness within the year preceding diagnosis; or incarceration at the time of TB diagnosis. Outbreaks were attributed to the jurisdiction in which >50% of outbreak-related cases were reported. SAS software (version 9.4; SAS Institute) was used for analysis. This activity was reviewed by CDC, deemed not research, and conducted consistent with applicable federal law and CDC policy.^§§^

## Results

### Characteristics of Large Outbreaks

During 2017–2023, a total of 50 large TB outbreaks were identified in the United States. An average of seven large outbreaks were identified per year, ranging from two in 2020 to 11 in 2018. These outbreaks included 1,092 cases (median = 18 cases per outbreak; range = 10–63) and occurred primarily in 23 states, including 17 with TB incidence below the national average of 2.6 cases per 100,000 population (Figure). TB cases in large outbreaks represented 1.7% of all TB cases reported during this period.

**FIGURE:**
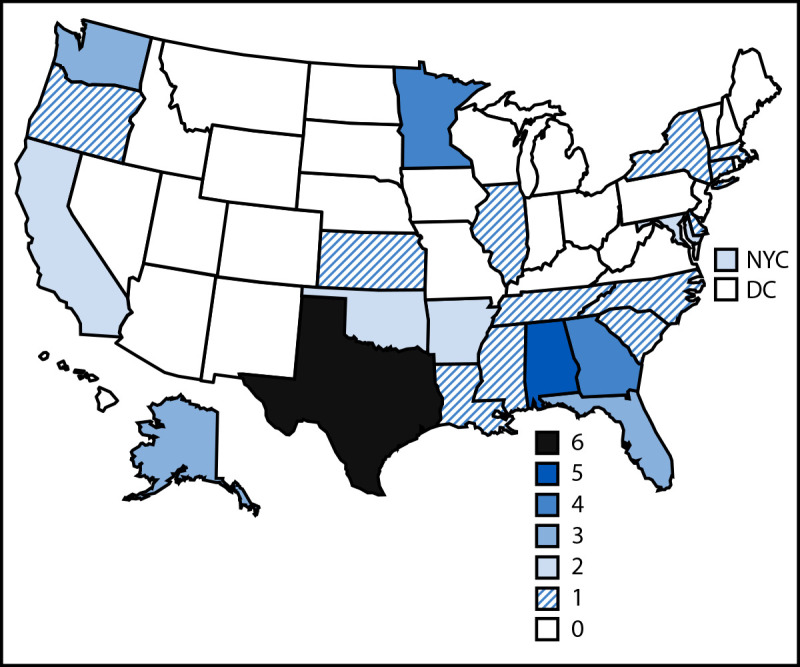
Large tuberculosis outbreaks,* by jurisdiction^†^ — United States, 2017–2023 **Abbreviations:** DC = District of Columbia; NYC = New York City. * N = 50. Ten or more cases of tuberculosis related by transmission within a 3-year period (*Mycobacterium tuberculosis* isolates differ by five or fewer single nucleotide polymorphisms or, when sequencing data are unavailable [e.g., for cases diagnosed without culture confirmation], an epidemiologic link to another outbreak-related case is present). ^†^ Outbreaks are attributed to the jurisdiction where >50% of outbreak-related cases were reported. NYC is a distinct reporting area from the state of New York.

### Characteristics of Persons with TB in Large Outbreaks

**Demographic characteristics.** Compared with 61,993 other persons who received a diagnosis of TB during 2017–2023, those who were part of large outbreaks were more frequently U.S.-born (79% versus 26%) (Table 1). They were also more frequently aged <15 years (15% versus 3%) or 25–44 years (40% versus 29%) and less frequently aged ≥65 years (8% versus 26%). Compared with TB cases not associated with large outbreaks, large outbreaks more frequently included U.S.-born persons identifying as non-Hispanic American Indian or Alaska Native (11% versus 1%) or non-Hispanic Black or African American (42% versus 9%), whereas large outbreaks less frequently included non–U.S.-born persons identifying as non-Hispanic Asian (5% versus 33%) or Hispanic or Latino (8% versus 24%).

**TABLE 1:** Characteristics of persons with tuberculosis in large outbreaks* compared with all other persons with tuberculosis — United States, 2017–2023

Characteristic	TB cases, no. (%)	
	In large outbreaks detected during 2017–2023^†^	Other cases reported during 2017–2023
**Total**	**1,092**	**61,993**
Male	728 (67)	37,809 (61)
**Age group, yrs**		
<15	165 (15)	1,787 (3)
15–24	163 (15)	6,001 (10)
25–44	436 (40)	18,245 (29)
45–64	243 (22)	18,380 (30)
≥65	85 (8)	16,144 (26)
Unknown	0 (—)	6 (1)
**Origin of birth^§^/Race and ethnicity^¶^**		
United States	866 (79)	15,956 (26)
AI/AN, NH	123 (11)	536 (1)
Asian, NH	9 (1)	857 (1)
Black or African American, NH	455 (42)	5,309 (9)
Hispanic or Latino	92 (8)	4,018 (7)
NH/PI, NH	44 (4)	517 (1)
White, NH	119 (11)	4,482 (7)
Multiple or other races, NH**	17 (2)	181 (1)
Unknown race and ethnicity	7 (1)	56 (1)
Outside the United States	226 (21)	45,644 (74)
AI/AN, NH	0 (—)	10 (1)
Asian, NH	51 (5)	20,141 (33)
Black or African American, NH	23 (2)	5,500 (9)
Hispanic or Latino	89 (8)	14,698 (24)
NH/PI, NH	49 (5)	2,604 (4)
White, NH	4 (1)	1,806 (3)
Multiple or other races, NH**	10 (1)	770 (1)
Unknown race and ethnicity	0 (—)	115 (1)
Unknown origin of birth	0 (—)	393 (1)
**Method of case detection**		
Contact tracing	292 (27)	2,653 (4)
Screening	78 (7)	5,596 (9)
TB signs or symptoms	509 (47)	36,430 (59)
Other^††^	209 (19)	16,814 (27)
Unknown	4 (1)	500 (1)
**Clinical signs of infectiousness**		
Pulmonary TB	968 (89)	49,685 (80)
Sputum smear–positive or cavitary TB	554 (51)	29,722 (48)
Among cases identified through contact tracing^§§^	67 (23)	576 (22)
Among cases not identified through contact tracing^¶¶^	485 (61)	28,987 (50)
Sputum smear–negative, noncavitary TB	358 (33)	24,357 (40)
Testing not performed or results unknown	180 (16)	7,914 (13)
**Other clinical characteristics**		
Completed TB treatment***	607 (92)	36,627 (94)
Deceased at diagnosis or died during treatment^†††^	43 (6)	4,343 (10)
Resistance to isoniazid or rifampin^§§§^	63 (7)	4,276 (9)
**TB risk factors**		
HIV coinfection	49 (5)	2,624 (4)
Non-HIV immunosuppression^¶¶¶^	51 (5)	4,204 (7)
Substance use ≤1 year before diagnosis****	297 (27)	7,706 (12)
Experienced homelessness ≤1 year before diagnosis	101 (9)	2,760 (5)
Resident of a correctional facility at diagnosis^††††^	122 (11)	1,722 (3)
Federal prison	0 (—)	224 (13)
State prison	89 (73)	244 (14)
Local jail	30 (25)	467 (27)
Other^§§§§^	2 (2)	766 (45)
Unknown	1 (1)	21 (1)
Resident of a long-term care facility at diagnosis	24 (2)	933 (2)

**Abbreviations**: AI/AN = American Indian or Alaska Native; NH = non-Hispanic; NH/PI = Native Hawaiian or Pacific Islander; TB = tuberculosis.

* Ten or more TB cases related by transmission within a 3-year period (*Mycobacterium tuberculosis* isolates differ by five or fewer single nucleotide polymorphisms or, when sequencing data are unavailable [e.g., for cases diagnosed without culture confirmation], an epidemiologic link to another outbreak-related case is present). This analysis only includes cases in large outbreaks that were initially detected during 2017–2023. Cases in large outbreaks that were detected before 2017 and were ongoing during the study period were grouped with all other TB cases.

^†^ Includes 81 cases that were reported during 2014–2016 and were part of large outbreaks that were detected during 2017–2019.

^§^ A person is considered U.S.-born if eligible for U.S. citizenship at birth, regardless of place of birth.

^¶^ Persons reporting Hispanic or Latino (Hispanic) ethnicity are categorized as Hispanic irrespective of race. NH persons are categorized by race.

** Includes NH persons who reported more than one race or a race not listed.

^††^ Includes other methods of case detection not listed, such as incidental chest radiograph findings, laboratory test results, or other unexpected clinical findings in which TB was not suspected at the time the test was ordered.

^§§^ Among 292 cases that were identified through contact tracing.

^¶¶^ Among 796 cases that were identified through screening, TB signs or symptoms, or other methods of case detection.

*** Patient completed the prescribed course of therapy. Numbers and percentages are based on patients who were alive at diagnosis and during treatment and had 2 years of follow-up (i.e., cases reported through 2021) so that treatment outcome could be documented. Denominators exclude 434 large outbreak-related cases and 23,182 cases that were not related to a large outbreak.

^†††^ Numbers and percentages are based on cases with 2 years of follow-up (i.e., cases reported through 2021) so that patient outcome could be documented. Denominators exclude 386 large outbreak-related cases and 18,337 cases that were not related to a large outbreak.

^§§§^ Resistance to at least isoniazid or rifampin on initial growth-based or molecular drug susceptibility testing. Percentage is calculated among cases for which growth-based or molecular drug susceptibility testing was performed. Denominators exclude 138 large outbreak-related cases and 14,894 cases that were not related to a large outbreak.

^¶¶¶^ Includes persons receiving tumor necrosis factor–alpha inhibitors at time of diagnostic evaluation, persons who have ever received a solid organ transplant, and persons who were immunocompromised at time of diagnostic evaluation because of a medical condition other than HIV or because of immunosuppressive therapy.

**** Substance use is defined as self-reported use of alcohol to excess, injection drugs, or noninjection drugs.

^††††^ Percentages are calculated among those who were incarcerated at the time of TB diagnosis.

^§§§§^ Includes other correctional facility types, such as juvenile detention centers, Immigration and Customs Enforcement detention centers, Indian reservation facilities, military stockades and jails, federal park police facilities, and police lockup facilities.

**Clinical and social characteristics.** The frequency of clinical features and medical risk factors for TB disease, including HIV coinfection, were similar among large outbreak-related and non–outbreak-related cases. However, social risk factors were more commonly reported among persons in large outbreaks than among all other persons with TB. These included substance use (27% versus 12%), homelessness (9% versus 5%), and incarceration (11% versus 3%). Similar percentages of persons with TB in large outbreaks and other persons with TB completed TB treatment (92% and 94%, respectively).

**Identification of large outbreak-related cases.** A total of 292 (27%) large outbreak-related cases were identified through contact tracing. Among these, a lower percentage had clinical markers of advanced, highly infectious disease (positive sputum smears or cavitary lung lesions) compared with large outbreak-related cases identified through other methods, including evaluation associated with TB symptoms, targeted testing or screening of persons at increased risk for TB, and incidental radiographic or laboratory findings suggestive of TB (23% versus 61%).

### Primary Settings and Networks of Transmission

Thirteen (26%) large outbreaks were primarily associated with congregate settings, including workplaces (five), correctional facilities (four), senior care facilities (two), a university (one), and a facility for persons experiencing homelessness (one) (Table 2). Thirty-four (68%) large outbreaks were primarily associated with family or social networks. In these outbreaks, transmission often occurred across multiple or unidentified settings, including private residences, community or social gatherings, or locations where substance use occurred. A primary setting or network of transmission could not be determined for three (6%) large outbreaks.

**TABLE 2:** Primary settings and networks of transmission* in large tuberculosis outbreaks^†^ — United States, 2017–2023

Setting and network	Large TB outbreaks, no. (%)^§^
**Total**	**50 (100)**
**Congregate setting**	13 (26)
Workplace	5 (10)
Correctional facility	4 (8)
Senior care facility	2 (4)
University	1 (2)
Facility for persons experiencing homelessness	1 (2)
**Family or social network**	34 (68)
Social network centered around substance use	8 (16)
Other familial or social network	26 (52)
**Unable to determine**	3 (6)

**Abbreviation:** TB = tuberculosis.

* Primary settings and networks of transmission were categorized using information obtained during routine communications with state and local health departments and were assigned based on the majority of outbreak-related cases.

**^†^** Ten or more TB cases related by transmission within a 3-year period (*Mycobacterium tuberculosis* isolates differ by five or fewer single nucleotide polymorphisms or, when sequencing data are unavailable [e.g., for cases diagnosed without culture confirmation], an epidemiologic link to another outbreak-related case is present).

^§^ Percentage calculated among all 50 outbreaks.

## Discussion

Despite low overall TB incidence in the United States, 50 large outbreaks involving 1,092 cases were identified during 2017–2023. Approximately 80% of large outbreak-related cases occurred among U.S.-born persons. The identification of large outbreaks in approximately one half of U.S. states, including many with TB incidence below the national average, indicates that maintaining public health capacity for TB outbreak prevention, detection, and response remains critical even in jurisdictions with low TB incidence.

Contact tracing is a key public health activity necessary for preventing and containing TB outbreaks. To prevent ongoing transmission, health departments systematically identify exposed contacts through patient interviews and review of administrative records. They also perform thorough medical evaluations and provide treatment to persons with TB disease and LTBI. The lower prevalence of markers of advanced, highly infectious disease among outbreak-related cases identified through contact tracing than among those identified through other methods, suggests that contact tracing might have prevented further transmission by facilitating earlier diagnosis and treatment when persons were less infectious.

Approximately one fourth of large TB outbreaks were associated with congregate settings; these settings can facilitate TB transmission because persons spend prolonged time in close proximity, often in crowded and poorly ventilated environments (*7*,*8*). Administrative infection control measures in overnight congregate settings, such as regular TB screening, maintaining resident rosters, and education for staff members and residents, are proven strategies for preventing the spread of TB (*5*,*7*–*9*). Large outbreaks in these settings, including one in a prison linked to disruptions in routine TB infection control practices (*4*), underscore the continued need for infection control measures in congregate settings, despite low overall U.S. TB incidence.

In contrast to transmission in congregate settings, approximately two thirds of large outbreaks were identified within family or social networks, where transmission occurred within private residences or across multiple or unidentified settings. Unlike congregate settings, where institutional records and policies can be used to focus outbreak response, identifying persons exposed to *M. tuberculosis* within family and social networks relies on the willingness and ability of persons with TB to provide names of contacts. Concerns about stigma, mistrust of government, involvement in illicit activities, and exposures in settings where identifying information is not exchanged can limit disclosure of contacts or participation in TB testing and treatment (*2*,*5*,*8*). Outbreak response in these situations often requires sustained efforts to build trust with affected persons and communities, such as forming partnerships with local cultural or religious institutions and community service providers, to identify and treat persons at risk (*2*–*5*).

Outreach can be more challenging when outbreaks involve substance use or homelessness; these situations were more common among persons in large outbreaks than among other persons with TB. These factors can increase risk for exposure and progression to TB disease while also complicating efforts to identify exposed persons and facilitate completion of TB treatment; this is because persons experiencing these situations might have unstable living conditions that can complicate follow-up, be unwilling or unable to name contacts, or have competing priorities that limit engagement with health care services (*5*,*8*). Strategies to overcome these barriers include mobile testing and treatment, shorter treatment regimens, and incentives and enablers (e.g., transportation, housing support, or food assistance) to support treatment adherence (*5*,*8*).

### Limitations

The findings in this report are subject to at least two limitations. First, large outbreaks are primarily identified using genomic data. Therefore, outbreaks with many clinically diagnosed cases without reported epidemiologic links might be missed, potentially resulting in an underestimate of large outbreaks. Second, because most persons infected with *M. tuberculosis* do not develop TB disease, and because LTBI is not nationally notifiable, the extent of outbreak-associated transmission described in this report is likely underestimated.

### Implications for Public Health Practice

Maintaining public health capacity for TB outbreak detection, prevention, and response remains essential, even in jurisdictions with low TB incidence. Continued national genomic surveillance is crucial to identifying and characterizing large outbreaks; at the same time, state and local TB programs should be prepared to expand routine TB control activities during outbreaks. Outbreak prevention and response strategies must also overcome barriers to diagnosis and treatment associated with homelessness and substance use. In addition, there is a need to build trust with affected persons and communities directly and through partnerships with local organizations and service providers. In congregate settings, maintaining procedures to promptly identify and isolate persons with infectious TB is critical for preventing TB outbreaks.

## Footnotes

* These authors contributed equally to this report.

† Universal whole genome sequencing of *M. tuberculosis* isolates from all persons with culture-positive TB disease began in 2018. Whole genome sequencing was performed for selected isolates submitted before 2018.

§ State and local health departments verify that TB cases reported to the National Tuberculosis Surveillance System meet the national surveillance case definition, which includes laboratory and clinical criteria.

¶ Defined as known or probable contact between two patients during either patient’s infectious period. The infectious period begins ≤3 months before symptom onset or TB diagnosis (whichever occurred earlier) and ends after ≥2 weeks of effective treatment, clinical improvement, and evidence of a microbiologic response (e.g., decrease in grade of sputum smear positivity).

** Because outbreaks are defined using a 3-year period, large outbreaks included in this report could include cases reported as early as 2014. Outbreaks ongoing as of 2023 were included, but any outbreak-related cases that were reported after 2023 were not included.

†† Defined as five or more drinks on the same occasion on ≥5 days during the past 30 days, participation in alcohol treatment or self-help programs, hospitalization for alcohol-related medical conditions, or more than one arrest for intoxication or drunk and disorderly behavior.

§§ 45 C.F.R. part 46.102(l)(2), 21 C.F.R. part 56; 42 U.S.C. Sect. 241(d); 5 U.S.C. Sect. 552a; 44 U.S.C. Sect. 3501 et seq.
